# Supplementary material for: A Planar-Type Micro-Biopsy Tool for a Capsule-Type Endoscope Using a One-Step Nickel Electroplating Process
Source: Micromachines (Basel). 2023 Oct 4;14(10):1900. doi: 10.3390/mi14101900 (PMC10609584; doi:10.3390/mi14101900)
Supplement: Supplementary file 1 [file micromachines-14-01900-s001.zip › micromachines-2620537-supplementary.pdf]

| | 10 mN vertical force | 30 mN vertical force | 0.3 N lateral force |
|---|---|---|---|
| Type 1 | 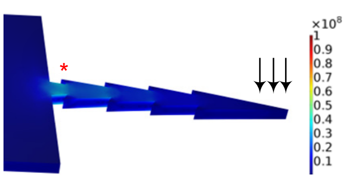 | 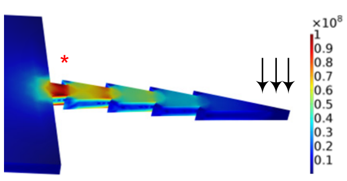 | 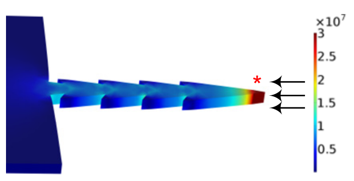 |
| Max | $3.49 \times 10^7$ N/m$^2$ | $1.05 \times 10^8$ N/m$^2$ | $3.36 \times 10^7$ N/m$^2$ |
| Type 2 | 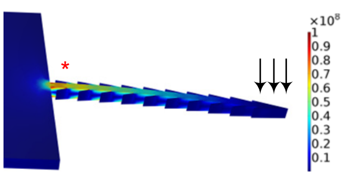 | 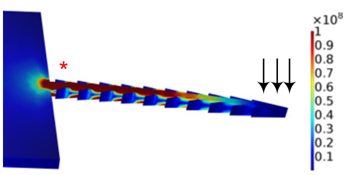 | 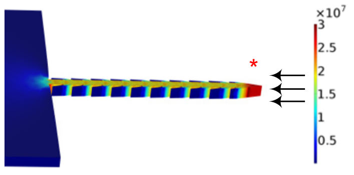 |
| | $9.96 \times 10^7$ N/m$^2$ | $2.99 \times 10^8$ N/m$^2$ | $3.78 \times 10^7$ N/m$^2$ |
| Type 3 | 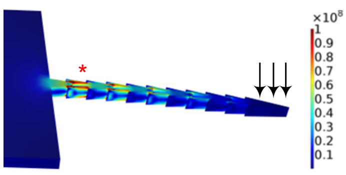 | 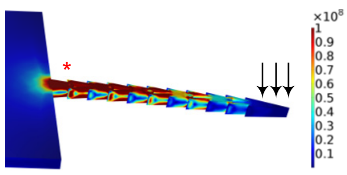 | 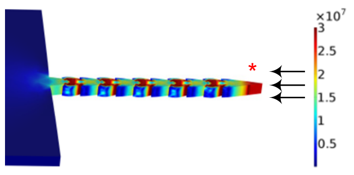 |
| Max | $16.9 \times 10^7$ N/m$^2$ | $5.08 \times 10^8$ N/m$^2$ | $8.41 \times 10^7$ N/m$^2$ |
| Type 4 | 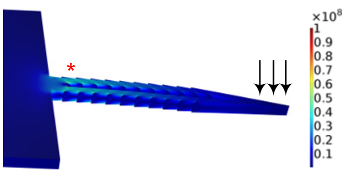 | 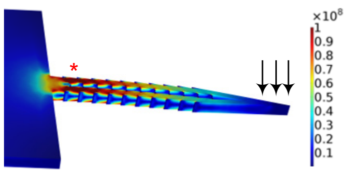 | 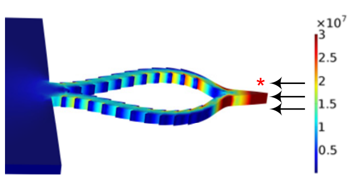 |
| Max | $6.41 \times 10^7$ N/m$^2$ | $1.92 \times 10^8$ N/m$^2$ | $4.58 \times 10^7$ N/m$^2$ |

**Figure S1.** Stress analysis of the designed micro-biopsy structure using a commercial simulation tool, COMSOL Multiphysics® modeling software, which enforced three different magnitudes of vertical force at the tip of the micro-biopsy tool for all types of designed micro-biopsy tool. Maximum Von Mises stress is shown at the end of the biopsy tip when the lateral force is applied and at the connection between the shank and base when the vertical force is applied, respectively.
